# M gene targeted qRT-PCR approach for SARS-CoV-2 virus detection

**DOI:** 10.1038/s41598-023-43204-9

**Published:** 2023-10-03

**Authors:** Md. Murshed Hasan Sarkar, Showti Raheel Naser, Sanjana Fatema Chowdhury, Md. Salim Khan, Md. Ahashan Habib, Shahina Akter, Tanjina Akhtar Banu, Barna Goswami, Iffat Jahan, Maksudur Rahman Nayem, Md. Akibul Hassan, Md. Imran Khan, Mohammad Fazle Alam Rabbi, Chowdhury Rafiqul Ahsan, Md. Ibrahim Miah, Afzalun Nessa, S. M. Rashed Ul Islam, Mohammed Atiqur Rahman, Md. Aftab Ali Shaikh, Md. Sharfuddin Ahmed

**Affiliations:** 1 Bangladesh Council of Scientific and Industrial Research (BCSIR), Dhaka, Bangladesh; 2 DNA Solution Ltd., Dhaka, Bangladesh; 3 University of Dhaka, Dhaka, Bangladesh; 4 Bangabandhu Sheikh Mujib Medical University (BSMMU), Dhaka, Bangladesh

**Keywords:** Microbiology, Molecular biology

## Abstract

Quantitative reverse transcriptase polymerase chain reaction (qRT-PCR) is the gold standard method for SARS-CoV-2 detection, and several qRT-PCR kits have been established targeting different genes of the virus. Due to the high mutation rate of these genes, false negative results arise thus complicating the interpretation of the diagnosis and increasing the need of alternative targets. In this study, an alternative approach for the detection of SARS-CoV-2 viral RNA targeting the membrane (M) gene of the virus using qRT-PCR was described. Performance evaluation of this newly developed in-house assay against commercial qRT-PCR kits was done using clinical oropharyngeal specimens of COVID-19 positive patients. The limit of detection was determined using successive dilutions of known copies of SARS-CoV-2 pseudovirus. The M gene based assay was able to detect a minimum of 100 copies of virus/mL indicating its capacity to detect low viral load. The assay showed comparable accuracy, sensitivity and specificity with commercially available kits while detecting all the variants efficiently. The study concluded that the in-house M gene based assay might be an effective alternative for the currently available commercial qRT-PCR kits.

## Introduction

The outbreak of coronavirus disease 2019 (COVID-19), caused by severe acute respiratory syndrome coronavirus 2 (SARS-CoV-2), was first detected at Wuhan, China in December 2019^1,2^. Since then, the virus has spread across the world, prompting the World Health Organization to designate it a pandemic (WHO, COVID-19 Situation Report-51, published) in March, 2020^3^. To date, more than 304 million people have been infected with SARS-CoV-2 and over 5.4 million souls were lost to COVID-19 globally^4^ and the number is increasing daily. Diagnosis is one of the most important pillars for pandemic response measures. High quality testing measures are critical for effective quarantine measures, travel restriction and contact tracing. Several factors are limiting the SARS-CoV-2 diagnosis such as high contagious rate and asymptomatic infections which warrants 40.5% of total infections. Thus, only clinical symptomatic diagnosis of COVID-19 is not effective against this pandemic and testing methods with higher sensitivity are of great importance^5,6^. Furthermore, adequate patient care and cleanliness protocols in hospitals are critical to limiting nosocomial spread among patients and healthcare workers^5^. The pandemic, however, poses unprecedented challenges for institutions performing diagnostic testing, as public health and patient care depend on rapid, reliable results.

Quantitative reverse transcriptase polymerase chain reaction (qRT-PCR) is the gold standard in the detection of SARS-CoV-2. Distinct qRT-PCR testing protocols were swiftly established and made publicly available by WHO^7^ and by the Center for Disease Control (CDC), USA^8^. To date, Food and Drug Administration (FDA), USA issued over 200 Emergency Use Authorization (EUA) COVID-19 molecular diagnostic kits for combating against this pandemic. However, many of these qRT-PCR kits exhibit lower sensitivity which creates possibility of providing false negative results on use. Moreover, other factors might also lead to false negative results, where low viral loads play a significant role^9–11^. Therefore, it is necessary to improve the analytical sensitivity to ensure the accuracy and reliability of the test results. Further, the specificity of the confirmatory test relies on the probe target sequence. The commercially available rRT-PCR kits generally target spike (S), nucleocapsid (N), envelope (E) or RNA-dependent RNA polymerase (RdRp) gene of SARS-CoV-2 already published by WHO. However, various mutations have been observed within these regions which might hamper sensitivity. Furthermore, several probes and primers are utilized in a multi-step PCR procedure in commercial kits, which is time-consuming and may complicate the result interpretation. Given the current scarcity of reagents, manpower, and equipment, a single gene qRT-PCR method with high sensitivity and specificity would be useful in combating the SARS-CoV-2 pandemic^5^. Optimized methods for both diagnostic and preemptive testing are vital for avoiding the worst-case scenario.

In search of a promising alternative, the M gene emerges as a viable choice despite its homology with SARS-CoV^12^, largely because of its lower mutation rate. It is reported that since the beginning of pandemic, M gene has acquired a ratio of missense to synonymous mutation below 1.0 and also accumulated less mutation than other gene (ORF1ab, S and N gene) used in commercial kits^13^. This makes M gene more suitable target then other genes.

In this study, an alternate RT-PCR approach specific for the detection of SARS-CoV-2 viral RNA was developed targeting the M gene. This approach involves designed primers binding to the M gene, which encodes the viral membrane protein. This novel primer pair exerted a better specificity and sensitivity than other FDA approved SARS-CoV-2 detection kit commercially available in Bangladesh. Local capacity build up and production strategies might further ensure continuous supply and price rationalization in developing countries like Bangladesh.

## Results

### Determination of M gene efficiency

The efficiency of the M gene was evaluated using true positive, false positive, true negative and false negative values (Supplementary File 1). The mathematical calculations demonstrated commendable sensitivity and specificity for M gene in comparison with other commercial SARS-CoV-2 detection kits (Table 1).

**Table 1:** Sensitivity and specificity of M gene kit in comparison with other three commercial detection kits. Table (A) denotes the True positive, True negative, False positive and False negative* value of M gene kit against three commercial kits. Table (B), (C), (D) denotes performance evaluation of M gene kit compared commercial kit 1, commercial kit 2, commercial kit 3 respectively for COVID-19 detection.

	M gene kit*					
(A) Commercial kit 1	Negative	Positive				
Negative	46	4				
Positive	2	68				
Commercial kit 2						
Negative	48	3				
Positive	0	69				
Commercial kit 3						
Negative	47	1				
Positive	1	71				

	Sensitivity % (95% CI)	Specificity % (95% CI)	PPV % (95% CI)	NPV % (95% CI)	Accuracy % (95% CI)	Kappa % (95% CI)
(B) Performance evaluation of M gene kit compared with commercial kit 1 for COVID-19 detection						
M gene kit vs commercial kit 1	97.14% (90.06–99.65%)	92.00% (80.77–97.78%)	94.44% (86.90–97.76%)	95.83% (85.41–98.91%)	95.00% (89.43–98.14%)	89.66% (81.59–97.72%)
(C) Performance evaluation of M gene kit compared with commercial kit 2 for COVID-19 detection						
M gene kit vs commercial kit 2	100% (94.79–100.00%)	94.12% (83.76–98.77%)	95.83% (88.47–98.57%)	100%	97.50% (92.87–99.48%)	94.85% (89.09–100%)
(D) Performance evaluation of M gene kit compared with commercial kit 3 for COVID-19 detection						
M gene kit vs commercial kit 3	98.61% (92.50–99.96%)	97.92% (88.93–99.95%)	98.61% (91.08–99.80%)	97.92% (87.03–99.70%)	98.33% (94.11–99.80%)	96.53% (91.76–100%)

*Here, positives and negatives were recognized according to the commercial kits.

### Determining the limit of detection (LOD) and qRT-PCR efficiency

**Figure 1:**
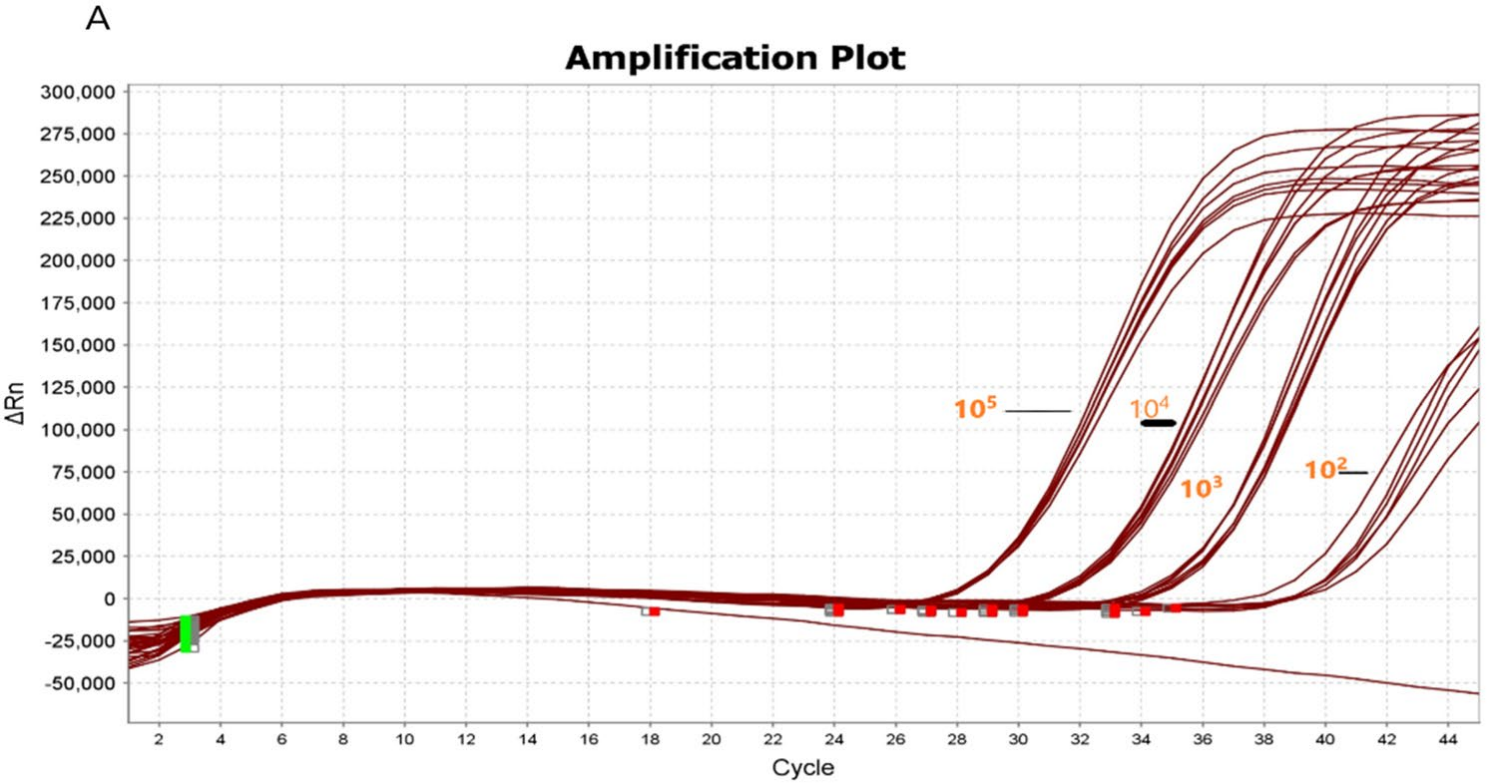
A serial dilution of pseudovirus containing SARS-CoV-2 viral genome (10^5^, 10^4^, 10^3^, 10^2^ and 10^1^ copies/mL) was prepared. Represents continuous amplification of all replicates of gradient dilution particles of pseudovirus containing SARS-CoV-2 genome.

To determine the LOD of M gene based assay for SARS-CoV-2 detection, serial dilution of pseudovirus RNA (10^5^, 10^4^, 10^3^, 10^2^ and 10^1^ copies/mL) was performed. All samples were tested with an M gene based kit to determine LOD (Fig. 1). It was observed that the M gene was able to identify samples with ≥ 100 copies/mL RNA with amplification efficiency (E) of 0.985 and regression coefficient of 0.985.

### Evaluation of sensitivity for different SARS-CoV-2 variants

**Figure 2:**
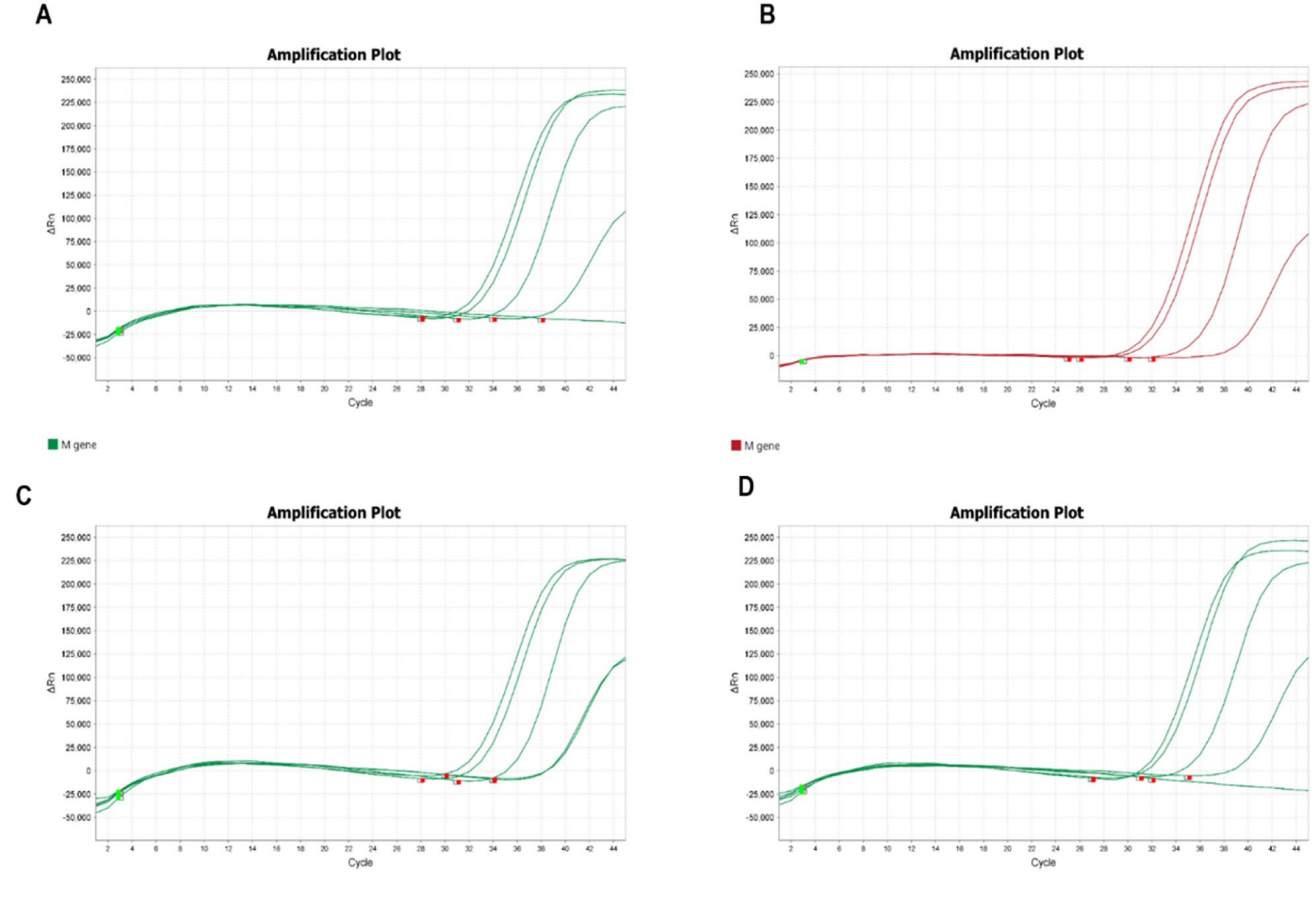
Differential dilutions of (15,000 copies/mL, 10,000 copies/mL, 100 copies/mL, 10 copies/mL) extracted variant reference materials were prepared. (**A**–**D**) Represent continuous amplification of Wuhan, UK, Brazil and African variant respectively.

The sensitivity of the M gene based assay has been evaluated against various SARS-CoV-2 variants. For this reason, pseudovirus RNA containing the specific mutation for Wuhan variant, UK variant, Brazil variant and African variant was used. Differentially diluted variant panel specific replicates (15,000 copies/mL, 10,000 copies/mL, 100 copies/mL, 10 copies/mL) were used. An M gene based assay considered in this study produced standard amplification curves up to 100 copies/mL for each variant (Fig. 2). However, it failed to recognize the samples containing 10 copies/mL of RNA. All the R^2^ scores of the experiments are given in Table 2.

**Table 2:** R^2^ values for variant specific experiments.

Sl. Nos.	SARS-CoV-2 Variants	R^2^ Score
1	Wuhan	0.770345288
2	Brazilian	0.899007784
3	UK	0.829460696
4	African	0.768066277

Moreover, nasopharyngeal samples that were previously sequenced and identified as Delta and Omicron variants (accession numbers ON909199, ON909200, ON909201, ON909202, ON909203, ON909204, ON909205, ON810538, ON810539, ON810540, ON810541, ON810542, ON810543, ON810544, ON980705, ON980706) were taken and this assay was also able to detect both variants successfully.

Along with that, the major variants of the SARS-CoV-2 virus were subjected to qRT-PCR, which showed comparable Ct values below the detectable range. The accession numbers of the variants are given in Supplementary File 2.

## Discussion

Countermeasures of any country against COVID-19 heavily rely on testing facilities with the highest possible sensitivity and specificity. Since the qRT-PCR method is regarded as the gold standard diagnosing the SARS-CoV-2 virus, it is the recommended diagnostic test for symptomatic and asymptomatic patients by WHO and CDC^14,15^. However, there are some drawbacks of qRT-PCR methods, which include the possibility of false negative or false positive results, high cost per test, etc.^16^. Several studies have been undertaken to date in trying to find a method for detecting the SARS-CoV-2 viral RNA with the highest sensitivity. To minimize possible false negative and positive results, developing primers with the best sensitivity to the target gene while avoiding cross-reactivity and optimizing qRT-PCR conditions are crucial. In commercially available kits, quantities and properties of chemicals and reagents (primers, probes, buffers, and enzymes) have been matched in such a way to reduce non-specific amplification^17^. However, in times of increasing reagent shortages, a simple protocol that can be quickly adapted with universal test kits and can be easily evaluated would be beneficial.

For COVID-19, the S, E, N, RdRP, and ORF1ab genes are widely used as target genes in qRT-PCR detection kits^18,19^. In China, kits targeting Orf1ab and N genes are regularly used, whereas N1, N2 and N3 genes are being utilized in US CDC^20^. Furthermore, in Europe, COVID-19 kits targeting E, N, and RdRP genes are frequently used^20^. An M gene is an important gene that codes for the M protein of SARS-CoV-2 virus. It is an integral membrane protein involved in several biological processes of the virus such as assembly, budding, matrix formation, and pathogenesis^21^. The primers and probe sets were designed for qRT-PCR to obtain the highest level of sensitivity against the target gene. In this study, M gene specific primers and probes were identified to be highly specific for the detection of SARS-CoV-2 viral RNA from oropharyngeal and nasopharyngeal swabs. According to the findings, the qRT-PCR efficiencies of tenfold dilution series of the standards were more than 99 for M gene, which matches the criteria for an efficient qRT-PCR assay^22,23^. Furthermore, the methodology enables the detection of SARS-CoV-2 viral RNA with a LOD value of 100 copies/mL for the M gene.

Due to the rapid evolution of SARS-CoV-2 genome^24^, the study focused on designing primers capable of covering all the variants of concern (VOC) such as alpha (B.1.1.7), Beta (B.1.351), Gamma (P.1), along with the most recent ones—Delta (B.1.617.2) and Omicron. That’s why, global SARS-CoV-2 genomes from various lineages and places were screened, and primer binding sites were identified as the most conserved regions. Then, considering the VOC of SARS-CoV-2, such as alpha, beta, gamma, delta and omicron, virus samples were also evaluated targeting M gene in this study. The LOD test demonstrated that M gene based qRT-PCR detected at least 100 copies/mL of the recent variant of SARS-CoV-2 virus. Moreover, the M gene based qRT-PCR works as a highly sensitive and specific molecular test to detect sub-lineages of Omicron variants. This work highlights the importance of constant monitoring of qRT-PCR to detect current circulating SARS-CoV-2 virus and reinforces M gene targeted qRT-PCR as a robust alternative for massive COVID-19 surveillance.

The commercial qRT-PCR kits can be performed basically consistently with the manufacturer's statement. The verification performance of the SARS-CoV-2 RNA test satisfied the assessment criteria, with a good result for the LOD, sensitivity, specificity and accuracy. The M gene based qRT-PCR assay was tested to verify its performance on clinical SARS-CoV-2 positive and negative samples. In addition, the results of the M gene were compared by using three commercial SARS-CoV-2 virus detection kits, namely commercial kit 1, commercial kit 2, and commercial kit 3. The sensitivity test of SARS-CoV-2 M gene assay versus commercial kit 1, commercial kit 2, and commercial kit 3 showed to be 97.14%, 100% and 98.61% respectively for SARS CoV-2 clinical samples (n = 220). On the other hand, specificity test for of SARS-CoV-2 M gene assay versus commercial kit 1, commercial kit 2, and commercial kit 3 showed to be 92%, 94.12% and 97.92% respectively. It is noteworthy that all the positive samples that were detected using M gene assay had Ct values that were around the cutoff value of 40.

In conclusion, M gene based PCR assay allows the quantification of very low viral loads having high sensitivity, specificity and accuracy for SARS-CoV-2 virus detection. This kit might be useful as an alternative to commercially available kits for not only diagnostics but also research purposes. Moreover, considering the sequence homology of M gene of SARS-CoV-2 and SARS-CoV viruses, studies can explore its potential to be used to detect other emerging coronaviruses in future.

## Materials and methods

### Consent for participation

Approval for ethical clearance (Ref: BMRC/NREC/2019–2022/109) was taken from Bangladesh Medical Research Council (BMRC). All methods were performed in accordance with the relevant guidelines and regulations of BMRC. Informed consent (both verbal and written) was obtained from all participants and/or their legal guardians for sample collection in Bangabandhu Sheikh Mujib Medical University (BSMMU), Dhaka and Centre for Advanced Research in Sciences (CARS), University of Dhaka, Dhaka.

### Primer design

**Figure 3:**
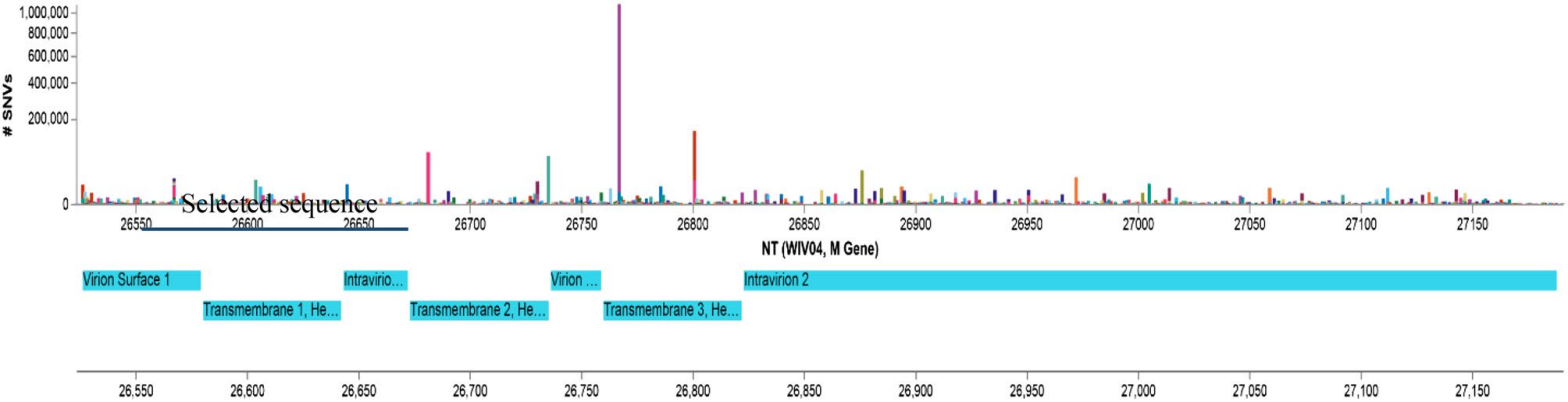
Mutation rate of M gene of SARS-CoV-2 virus.

M gene was selected as the target for primer designing due to its low mutation rate (a ratio of missense to synonymous mutation below 1.0)^13^ (Fig. 3). Primers were designed based on the recently available complete genome sequences of SARS-CoV-2 virus from the Global Initiative on Sharing All Influenza Data (GISAID) Reference Sequence Database (https://www.epicov.org/epi3). Integrated DNA Technology (IDT) PrimerQuest Tool (https://sg.idtdna.com/pages/tools/primerquest) was utilized to design specific primers and probe (Table 3). The primer sets were synthesized and supplied by IDT (USA) according to our proposed design. A standard PCR was performed using three randomly chosen COVID-19 positive samples in order to validate the product size of the designed primer sets (Supplementary file 3).

**Table 3:** Designed primer–probe for M gene.

Oligonucleotides for M gene	Sequence (5′–3′)	Length (nt)
Forward primer	GGT ACT ATT ACC GTT GAA GAG CTT A	25
Reverse primer	CTG TTG GCA TAG GCA AAT TGT AG	25
Probe	5Cy5/AG CTC CTT GAA CAA TGG AAC CTA GT/3BHQ_2	23

### Sample collection

Nasopharyngeal or oropharyngeal swabs were collected from patients suspected of having SARS-CoV-2 at Bangabandhu Sheikh Mujib Medical University (BSMMU) and Centre for Advanced Research in Sciences (CARS), University of Dhaka. In order to validate accuracy of the kit, negative samples were also collected. The samples were placed in BD Universal Transport Medium (UTM) (Becton, Dickinson and Company, USA) and transported to the laboratory Bangladesh Council of Scientific and Industrial Research (BCSIR) by maintaining the cold chain for further processing.

### RNA extraction methods evaluation

RNA extraction was performed using ReliaPrep™ Viral Total Nucleic Acid Purification Kit (Promega, Madison, USA, Cat.# AX4820) as per the manufacturer’s protocols. For each extraction, 200 µL of sample was used and eluted in 60 µL nuclease-free water.

### Quantitative reverse transcription polymerase chain reaction (qRT-PCR) using commercial and M gene based kit

In order to validate the M gene based assay, 220 random oropharyngeal clinical specimens suspected with SARS-CoV-2 were selected using commercial CE-IVD certified kits. qRT-PCR was performed with all samples simultaneously with the developed M gene based kit and other commercially available kits denoted as commercial kit 1, commercial kit 2 and commercial kit 3 following manufacturer’s instructions. For M gene based kit, GoTaq Probe 1-Step RT-PCR master mix (Promega Corporation, Madison, USA) was used. In brief, each reaction consisted of a total volume of 20 µL containing 1 µL of the designed primer and probe mixture (10 pM/µL), 5 µL of viral RNA, 10 µL dUTP, 0.4 µL Reverse transcriptase enzyme and 3.6 µL of RNase-free water. Real-time PCR was performed using qTower Real-Time PCR Machine (Analytikjena, Germany). The thermal cycler conditions were set as follows: 45 °C for 15 min, 95 °C for 2 min, followed by 45 cycles of amplification at 94 °C for 10 s, 52 °C for 15 s and 60 °C for 30 s. The reaction was completed by determining the dissociation curve of all amplicons. Cy5 was selected as the detection dye for this reaction.

### Limit of detection (LOD) determination

To determine the limit of detection (LOD) of the in-house one step SARS-CoV-2 qRT-PCR assay, positive reference materials of SARS-CoV-2 pseudovirus were used (AccuPlexTM SARS-CoV-2 Verification Panel Full Genome, LGC SeraCare, USA). The reference materials contained known concentrations of virus particles which were serially diluted starting from 100,000 copies/mL to 1 copy/mL. RNA was isolated according to the previously described method (see the above RNA extraction procedure) and used as the template.

### Specific variant determination using an M gene based kit

Since the beginning of the pandemic, BCSIR has been sequencing SARS-CoV-2 virus sequences for different research purposes. From the obtained sequenced data, major SARS-CoV-2 variants (alpha, beta, gamma, delta and omicron) were isolated and used as templates for checking the efficiency of the M gene based kit for variant detection. In addition, the LOD of the in-house one step SARS-CoV-2 qRT-PCR assay was determined against the SARS-CoV-2 viral RNA genome variant panel representing prominent variants (Alpha, Beta, Gamma, Delta and Omicron) and wild type (Wuhan) variant (AccuplexTM SARS-CoV-2 Variant Panel 1, LGC SeraCare, USA). The variant reference materials were differentially diluted (100,000 copies/mL, 10,000 copies/mL, 1000 copies/mL, 100 copies/mL, 10 copies/mL, and 1 copy/mL).

### Statistical analysis for determining the efficiency of the kit

Fisher’s exact test was done to compare the efficiency of the in-house kit and other commercial COVID-19 detection kits. Determining the sensitivity, specificity, Positive Predictive Value (PPV), and Negative Predictive Value (NPV) are crucial for establishing any diagnostic protocol or kit. These values were calculated using the following formula.

$$\mathrm{Sensitivity }=\frac{\text{True Positive}}{\text{True Positive + False Negative}}$$

$$\mathrm{Specificity }=\frac{\text{True Negative}}{\text{True Negative + False Positive}}$$

$$\mathrm{Positive Predictive Value }(\mathrm{PPV}) =\frac{\text{True Positive}}{\text{True Positive + False Positive}}$$

$$\mathrm{Negative Predictive Value }(\mathrm{NPV}) =\frac{\text{True Negative}}{\text{True Negative + False Negative}}$$

### Supplementary Information

Supplementary Table 1.

Supplementary Table 2.

Supplementary Figures.

## Data Availability

Primer and probe sequences related to this study are available within this manuscript. The whole genome sequences used for the method validation are available in the NCBI database. The accession numbers are added to the supplementary file 2. The method evaluation data is available in supplementary file 1. The product size evaluation gel image and the overall flow chart are available in supplementary file 3.
