# Supplementary material for: M gene targeted qRT-PCR approach for SARS-CoV-2 virus detection
Source: Sci Rep. 2023 Oct 3;13:16659. doi: 10.1038/s41598-023-43204-9 (PMC10547753; doi:10.1038/s41598-023-43204-9)
Supplement: Supplementary file 1 — Supplementary Table 1. [file 41598_2023_43204_MOESM1_ESM.docx]

**Supplementary File 01**

**Table-01: Comparative Ct values of 220 clinical specimens with different kits commercial kit -1, commercial kit-2, commercial kit-3**

| Sample ID | M-gene Kit | Commercial kit 2 | | | Commercial kit 1 | | Commercial kit 3 | | Comments |
| --- | --- | --- | --- | --- | --- | --- | --- | --- | --- |
|  | M gene (CT value) | E (CT value) | N (CT value) | Rdrp(CT value) | N(CT value) | ORF(CT value) | E (CT value) | Rdrp(CT value) |  |
| 17 | 22.287 | 19.9 | 19.68 | 20.67 | 20.52 | 23.34 | 19.9 | 20.67 | Positive |
| 22 | 26.218 | 24.33 | 23.3 | 24.8 | 20.62 | 22.05 | 24.33 | 24.8 | Positive |
| 26 | 27.272 | 25.6 | 24.76 | 25.55 | 20.93 | 24.15 | 25.6 | 25.55 | Positive |
| 28 | 26.618 | 25.48 | 23.41 | 25.69 | 21.15 | 24.31 | 25.48 | 25.69 | Positive |
| 57 | 32.135 | 30.63 | 30.1 | 30.18 | 21.17 | 23.26 | 30.63 | 30.18 | Positive |
| 85 | 16.471 | 15.37 | 15.52 | 16.11 | 21.26 | 23.41 | 15.37 | 16.11 | Positive |
| 107 | 21.816 | 19.83 | 19.55 | 20.45 | 21.31 | 23.96 | 19.83 | 20.45 | Positive |
| 439 | 22.607 | 20.76 | 20.69 | 21.85 | 21.32 | 24.32 | 20.76 | 21.85 | Positive |
| 22 | 17.408 | 15.73 | 15.84 | 16.38 | 22.06 | 21.23 | 15.73 | 16.38 | Positive |
| 33 | 31.216 | 29.59 | 28.99 | 29.68 | 22.11 | 23.83 | 29.59 | 29.68 | Positive |
| 41 | 24.051 | 22.11 | 21.58 | 23 | 22.28 | 23.4 | 22.11 | 23 | Positive |
| 57 | 32.976 | 31.35 | 28.92 | 32.6 | 22.42 | 26.89 | 31.35 | 32.6 | Positive |
| 8 | 22.231 | 20.27 | 19.98 | 21.02 | 22.68 | 25.01 | 20.27 | 21.02 | Positive |
| 33 | 25.28 | 23.89 | 32.91 | 20.98 | 22.28 | 23.4 | 24.78 | 22.58 | Positive |
| 46 | 26.93 | 25.28 | 31.14 | 21.81 | 22.42 | 26.89 | 22.49 | 21.09 | Positive |
| 55 | 25.21 | 28.82 | 37.16 | 24.78 | 22.64 | 25.51 | 28.37 | 27.57 | Positive |
| 50 | 29.336 | 27.96 | 26.95 | 27.83 | 20.27 | 25.09 | 27.96 | 27.83 | Positive |
| 51 | No Ct | No Ct | No Ct | No Ct | No Ct | No Ct | No Ct | No Ct | Negative |
| 52 | No Ct | No Ct | No Ct | No Ct | No Ct | No Ct | No Ct | No Ct | Negative |
| 55 | 18.056 | 16.58 | 16.6 | 17 | 20.27 | 23.33 | 16.58 | 17 | Positive |
| 60 | 29.336 | 27.96 | 26.95 | 27.83 | 20.27 | 25.09 | 27.96 | 27.83 | Positive |
| 61 | 16.249 | 14.96 | 14.91 | 15.58 | 20.38 | 24.02 | 14.96 | 15.58 | Positive |
| 62 | No Ct | No Ct | No Ct | No Ct | No Ct | No Ct | No Ct | No Ct | Negative |
| 63 | No Ct | No Ct | No Ct | No Ct | No Ct | No Ct | No Ct | No Ct | Negative |
| 64 | No Ct | No Ct | No Ct | No Ct | No Ct | No Ct | No Ct | No Ct | Negative |
| 65 | No Ct | No Ct | No Ct | No Ct | No Ct | No Ct | No Ct | No Ct | Negative |
| 74 | 27.69 | 26.31 | 31.84 | 26.64 | 31.84 | 32.25 | 26.31 | 26.64 | Positive |
| 75 | No Ct | No Ct | No Ct | No Ct | No Ct | No Ct | No Ct | No Ct | Negative |
| 76 | No Ct | No Ct | No Ct | No Ct | No Ct | No Ct | No Ct | No Ct | Negative |
| 77 | 33.62 | 31.94 | 38.66 | 32.35 | 38.66 | 39.16 | 31.94 | 32.35 | Positive |
| 89 | 28.84 | 28.29 | 34.57 | 26.23 | 22.68 | 25.01 | 35.05 | 35.55 | Positive |
| 104 | 37.06 | 30.56 | 29.77 | 30.95 | 29.39 | 33.83 | 30.18 | 30.56 | Positive |
| 421 | 32.85 | 23.71 | 29.77 | 24.01 | 29.39 | 36.38 | 23.41 | 23.71 | Positive |
| 147 | 31.18 | 34.26 | 29.93 | 34.7 | 29.55 | 34.83 | 33.83 | 34.26 | Positive |
| 158 | 30.18 | 29.74 | 31.31 | 30.12 | 30.91 | 35.26 | 29.37 | 29.74 | Positive |
| 12 | 26.676 | 25.5 | 25.42 | 25.73 | 21.7 | 26.54 | 25.5 | 25.73 | Positive |
| 14 | No Ct | No Ct | No Ct | No Ct | No Ct | No Ct | No Ct | No Ct | Negative |
| 15 | No Ct | No Ct | No Ct | No Ct | No Ct | No Ct | No Ct | No Ct | Negative |
| 35 | 23.492 | 21.7 | 21.58 | 22.54 | 21.87 | 24.25 | 21.7 | 22.54 | Positive |
| 49 | 28.914 | 27.27 | 26.99 | 27.26 | 21.93 | 25.15 | 27.27 | 27.26 | Positive |
| 51 | No Ct | No Ct | No Ct | No Ct | No Ct | No Ct | No Ct | No Ct | Negative |
| 52 | No Ct | No Ct | No Ct | No Ct | No Ct | No Ct | No Ct | No Ct | Negative |
| 53 | No Ct | No Ct | No Ct | No Ct | No Ct | No Ct | No Ct | No Ct | Negative |
| 55 | No Ct | No Ct | No Ct | No Ct | 30.67 | 35.25 | No Ct | No Ct | Negative/ Positive |
| 56 | No Ct | No Ct | No Ct | No Ct | 30.7 | 33.73 | No Ct | No Ct | Negative/ Positive |
| 57 | No Ct | No Ct | No Ct | No Ct | No Ct | No Ct | No Ct | No Ct | Negative |
| 58 | No Ct | No Ct | No Ct | No Ct | No Ct | No Ct | No Ct | No Ct | Negative |
| 59 | 18.056 | 16.58 | 16.6 | 17 | 20.27 | 23.33 | 16.58 | 17 | Positive |
| 1 | No Ct | No Ct | No Ct | No Ct | No Ct | No Ct | No Ct | No Ct | Negative |
| 2 | No Ct | No Ct | No Ct | No Ct | No Ct | No Ct | No Ct | No Ct | Negative |
| 3 | No Ct | No Ct | No Ct | No Ct | No Ct | No Ct | No Ct | No Ct | Negative |
| 4 | No Ct | No Ct | No Ct | No Ct | No Ct | No Ct | No Ct | No Ct | Negative |
| 73 | 22.633 | 21.34 | 18.65 | 23.46 | 22.64 | 25.51 | 21.34 | 23.46 | Positive |
| 74 | No Ct | No Ct | No Ct | No Ct | No Ct | No Ct | No Ct | No Ct | Negative |
| 75 | No Ct | No Ct | No Ct | No Ct | No Ct | No Ct | No Ct | No Ct | Negative |
| 76 | No Ct | No Ct | No Ct | No Ct | No Ct | No Ct | No Ct | No Ct | Negative |
| 1 | No Ct | No Ct | No Ct | No Ct | No Ct | No Ct | No Ct | No Ct | Negative |
| 2 | No Ct | No Ct | No Ct | No Ct | No Ct | No Ct | No Ct | No Ct | Negative |
| 3 | No Ct | No Ct | No Ct | No Ct | No Ct | No Ct | No Ct | No Ct | Negative |
| 4 | 28.84 | 28.29 | 34.57 | 26.23 | 22.68 | 25.01 | 35.05 | 35.55 | Positive |
| 26 | 30.68 | 21.64 | 30.13 | 21.92 | 29.75 | 33.84 | 21.37 | 21.64 | Positive |
| 27 | No Ct | No Ct | No Ct | No Ct | No Ct | No Ct | No Ct | No Ct | Negative |
| 28 | No Ct | No Ct | No Ct | No Ct | No Ct | No Ct | No Ct | No Ct | Negative |
| 29 | 26.93 | 25.28 | 31.14 | 21.81 | 22.42 | 26.89 | 22.49 | 21.09 | Positive |
| 30 | No Ct | No Ct | No Ct | No Ct | No Ct | No Ct | No Ct | No Ct | Negative |
| 31 | No Ct | No Ct | No Ct | No Ct | No Ct | No Ct | No Ct | No Ct | Negative |
| 32 | 28.02 | 28.84 | 32.72 | 29.21 | 32.31 | 33.93 | 28.48 | 28.84 | Positive |
| 43 | 33.62 | 29.74 | 32.92 | 30.12 | 32.51 | 38.99 | 29.37 | 29.74 | Positive |
| 44 | No Ct | No Ct | No Ct | No Ct | No Ct | No Ct | No Ct | No Ct | Negative |
| 45 | No Ct | No Ct | No Ct | No Ct | No Ct | No Ct | No Ct | No Ct | Negative |
| 46 | No Ct | No Ct | No Ct | No Ct | No Ct | No Ct | No Ct | No Ct | Negative |
| 47 | No Ct | No Ct | No Ct | No Ct | No Ct | No Ct | No Ct | No Ct | Negative |
| 48 | 21.84 | 15.64 | 31.22 | 15.84 | 30.82 | 33.94 | 15.44 | 15.64 | Positive |
| 50 | 36.11 | 28.84 | 31.29 | 29.21 | 30.9 | 34.74 | 28.48 | 28.84 | Positive |
| 51 | No Ct | No Ct | No Ct | No Ct | No Ct | No Ct | No Ct | No Ct | Negative |
| 57 | 33.7 | 23.39 | 30.28 | 23.69 | 29.9 | 31.98 | 23.1 | 23.39 | Positive |
| 60 | 32.93 | 34.21 | 30.74 | 34.65 | 30.35 | 35.02 | 33.78 | 34.21 | Positive |
| 61 | No Ct | No Ct | No Ct | No Ct | No Ct | No Ct | No Ct | No Ct | Negative |
| 62 | No Ct | No Ct | No Ct | No Ct | No Ct | No Ct | No Ct | No Ct | Negative |
| 63 | 16.249 | 14.96 | 14.91 | 15.58 | 20.38 | 24.02 | 14.96 | 15.58 | Positive |
| 65 | 22.287 | 19.9 | 19.68 | 20.67 | 20.52 | 23.34 | 19.9 | 20.67 | Positive |
| 66 | 32.18 | No Ct | No Ct | 34.64 | No Ct | No Ct | 35.92 | 32.56 | Negative/ Positive |
| 67 | No Ct | No Ct | No Ct | No Ct | No Ct | No Ct | No Ct | No Ct | Negative |
| 68 | No Ct | No Ct | No Ct | No Ct | No Ct | No Ct | No Ct | No Ct | Negative |
| 70 | No Ct | No Ct | No Ct | No Ct | No Ct | No Ct | No Ct | No Ct | Negative |
| 71 | 38.46 | No Ct | No Ct | No Ct | No Ct | No Ct | No Ct | No Ct | Positive/ Negative |
| 72 | No Ct | No Ct | No Ct | No Ct | No Ct | No Ct | No Ct | No Ct | Negative |
| 73 | 31.31 | 30.37 | 31.65 | 26.8 | No Ct | No Ct | 28.62 | 25.57 | Negative/ Positive |
| 75 | No Ct | No Ct | No Ct | No Ct | No Ct | No Ct | No Ct | No Ct | Negative |
| 76 | No Ct | No Ct | No Ct | 30.87 | No Ct | No Ct | 33.24 | 30.47 | Negative/ Positive |
| 85 | 29.3 | 22.73 | 32.47 | 23.02 | 32.06 | 34.55 | 22.44 | 22.73 | Positive |
| 95 | 32.11 | 25.3 | 32.12 | 25.62 | 31.71 | 35.16 | 24.98 | 25.3 | Positive |
| 1 | No Ct | No Ct | No Ct | No Ct | No Ct | No Ct | No Ct | No Ct | Negative |
| 2 | No Ct | No Ct | No Ct | No Ct | No Ct | No Ct | No Ct | No Ct | Negative |
| 3 | No Ct | No Ct | No Ct | No Ct | No Ct | No Ct | No Ct | No Ct | Negative |
| 4 | No Ct | No Ct | No Ct | No Ct | No Ct | No Ct | No Ct | No Ct | Negative |
| 51 | 31.72 | 25.56 | 31.87 | 25.89 | 31.47 | 38.48 | 25.24 | 25.56 | Positive |
| 52 | No Ct | No Ct | No Ct | No Ct | No Ct | No Ct | No Ct | No Ct | Negative |
| 53 | No Ct | No Ct | No Ct | No Ct | No Ct | No Ct | No Ct | No Ct | Negative |
| 54 | No Ct | No Ct | No Ct | No Ct | No Ct | No Ct | No Ct | No Ct | Negative |
| 55 | 26.618 | 25.48 | 23.41 | 25.69 | 21.15 | 24.31 | 25.48 | 25.69 | Positive |
| 62 | 32.135 | 30.63 | 30.1 | 30.18 | 21.17 | 23.26 | 30.63 | 30.18 | Positive |
| 63 | 30.35 | 30.41 | 32.2 | 30.8 | 31.8 | 36.47 | 30.03 | 30.41 | Positive |
| 17 | 26.19 | 18.96 | 32.39 | 19.2 | 31.99 | 30.78 | 18.72 | 18.96 | Positive |
| 17 | 24.69 | 18.91 | 32.72 | 19.15 | 32.31 | 33.93 | 18.67 | 18.91 | Positive |
| 28 | 33.61 | 33.04 | 32.92 | 33.46 | 32.51 | 38.99 | 32.62 | 33.04 | Positive |
| 29 | No Ct | No Ct | No Ct | No Ct | No Ct | No Ct | No Ct | No Ct | Negative |
| 30 | No Ct | No Ct | No Ct | No Ct | No Ct | No Ct | No Ct | No Ct | Negative |
| 31 | 21.816 | 19.83 | 19.55 | 20.45 | 21.31 | 23.96 | 19.83 | 20.45 | Positive |
| 33 | 32.19 | 32.8 | 33.25 | 33.22 | 32.83 | 36.99 | 32.39 | 32.8 | Positive |
| 34 | No Ct | No Ct | No Ct | No Ct | No Ct | No Ct | No Ct | No Ct | Negative |
| 35 | No Ct | No Ct | No Ct | No Ct | No Ct | No Ct | No Ct | No Ct | Negative |
| 36 | 16.471 | 15.37 | 15.52 | 16.11 | 21.26 | 23.41 | 15.37 | 16.11 | Positive |
| 37 | No Ct | No Ct | No Ct | No Ct | No Ct | No Ct | No Ct | No Ct | Negative |
| 38 | No Ct | No Ct | No Ct | No Ct | No Ct | No Ct | No Ct | No Ct | Negative |
| 39 | No Ct | No Ct | No Ct | No Ct | No Ct | No Ct | No Ct | No Ct | Negative |
| 40 | 27.69 | 30.41 | 33.31 | 30.8 | 31.79 | 35.16 | 30.03 | 30.41 | Positive |
| 49 | 32.47 | 25.8 | 33.31 | 26.13 | 32.89 | 36.26 | 25.48 | 25.8 | Positive |
| 50 | 28.02 | 28.84 | 32.72 | 29.21 | 32.31 | 33.93 | 28.48 | 28.84 | Positive |
| 53 | 33.62 | 29.74 | 32.92 | 30.12 | 32.51 | 38.99 | 29.37 | 29.74 | Positive |
| 55 | No Ct | No Ct | No Ct | No Ct | No Ct | No Ct | No Ct | No Ct | Negative |
| 56 | No Ct | No Ct | No Ct | No Ct | No Ct | No Ct | No Ct | No Ct | Negative |
| 59 | 28.79 | 25.3 | 33.31 | 25.62 | 32.89 | 36.26 | 24.98 | 25.3 | Positive |
| 61 | 23.14 | 25.56 | 33.25 | 25.89 | 32.83 | 36.99 | 25.24 | 25.56 | Positive |
| 62 | No Ct | No Ct | No Ct | No Ct | No Ct | No Ct | No Ct | No Ct | Negative |
| 74 | 27.65 | 26.27 | 31.8 | 26.6 | 31.8 | 32.2 | 26.27 | 26.6 | Positive |
| 76 | 30.62 | 29.09 | 35.21 | 29.46 | 35.21 | 35.66 | 29.09 | 29.46 | Positive |
| 78 | 30.51 | 28.98 | 35.09 | 29.35 | 35.09 | 35.53 | 28.98 | 29.35 | Positive |
| 80 | 28.37 | 26.95 | 32.63 | 27.3 | 32.63 | 33.04 | 26.95 | 27.3 | Positive |
| 84 | No Ct | No Ct | No Ct | No Ct | No Ct | No Ct | No Ct | No Ct | Negative |
| 85 | No Ct | No Ct | No Ct | No Ct | No Ct | No Ct | No Ct | No Ct | Negative |
| 86 | No Ct | No Ct | No Ct | No Ct | No Ct | No Ct | No Ct | No Ct | Negative |
| 88 | 29.58 | 28.1 | 34.02 | 28.46 | 34.02 | 34.45 | 28.1 | 28.46 | Positive |
| 18 | 25.77 | 24.48 | 29.64 | 24.79 | 29.64 | 30.01 | 24.48 | 24.79 | Positive |
| 107 | 26.15 | 24.84 | 30.07 | 25.16 | 30.07 | 30.46 | 24.84 | 25.16 | Positive |
| 1 | No Ct | No Ct | No Ct | No Ct | No Ct | No Ct | No Ct | No Ct | Negative |
| 2 | No Ct | No Ct | No Ct | No Ct | No Ct | No Ct | No Ct | No Ct | Negative |
| 3 | No Ct | No Ct | No Ct | No Ct | No Ct | No Ct | No Ct | No Ct | Negative |
| 4 | No Ct | No Ct | No Ct | No Ct | No Ct | No Ct | No Ct | No Ct | Negative |
| 5 | 23.98 | 22.78 | 27.58 | 23.07 | 27.58 | 27.93 | 22.78 | 23.07 | Positive |
| 39 | 27.54 | 26.16 | 31.67 | 26.5 | 31.67 | 32.07 | 26.16 | 26.5 | Positive |
| 40 | No Ct | No Ct | No Ct | No Ct | No Ct | No Ct | No Ct | No Ct | Negative |
| 41 | No Ct | No Ct | No Ct | No Ct | No Ct | No Ct | No Ct | No Ct | Negative |
| 42 | No Ct | No Ct | No Ct | No Ct | No Ct | No Ct | No Ct | No Ct | Negative |
| 43 | No Ct | No Ct | No Ct | No Ct | No Ct | No Ct | No Ct | No Ct | Negative |
| 44 | No Ct | No Ct | No Ct | No Ct | No Ct | No Ct | No Ct | No Ct | Negative |
| 45 | 25.21 | 28.82 | 37.16 | 24.78 | 22.64 | 25.51 | 28.37 | 27.57 | Positive |
| 50 | 23.12 | 21.96 | 26.59 | 22.24 | 26.59 | 26.93 | 21.96 | 22.24 | Positive |
| 51 | No Ct | No Ct | No Ct | No Ct | No Ct | No Ct | No Ct | No Ct | Negative |
| 52 | No Ct | No Ct | No Ct | No Ct | No Ct | No Ct | No Ct | No Ct | Negative |
| 54 | 28.02 | 26.62 | 32.22 | 26.96 | 32.22 | 32.63 | 26.62 | 26.96 | Positive |
| 55 | 33.62 | 31.94 | 38.66 | 32.35 | 38.66 | 39.16 | 31.94 | 32.35 | Positive |
| 56 | No Ct | No Ct | No Ct | No Ct | No Ct | No Ct | No Ct | No Ct | Negative |
| 59 | 26.3 | 24.99 | 30.25 | 25.3 | 30.25 | 30.63 | 24.99 | 25.3 | Positive |
| 60 | 26.15 | 24.84 | 30.07 | 25.16 | 30.07 | 30.46 | 24.84 | 25.16 | Positive |
| 62 | 26.54 | 25.21 | 30.52 | 25.53 | 30.52 | 30.91 | 25.21 | 25.53 | Positive |
| 73 | 27.42 | 26.05 | 31.53 | 26.38 | 31.53 | 31.94 | 26.05 | 26.38 | Positive |
| 77 | 31.82 | 30.23 | 36.59 | 30.61 | 36.59 | 37.06 | 30.23 | 30.61 | Positive |
| 78 | 30.07 | 28.57 | 34.58 | 28.93 | 34.58 | 35.02 | 28.57 | 28.93 | Positive |
| 80 | No Ct | No Ct | No Ct | No Ct | No Ct | No Ct | No Ct | No Ct | Negative |
| 81 | No Ct | No Ct | No Ct | No Ct | No Ct | No Ct | No Ct | No Ct | Negative |
| 82 | No Ct | No Ct | No Ct | No Ct | No Ct | No Ct | No Ct | No Ct | Negative |
| 2 | 23.14 | 21.98 | 26.61 | 22.26 | 26.61 | 26.95 | 21.98 | 22.26 | Positive |
| 4 | 28.79 | 27.35 | 33.11 | 27.7 | 33.11 | 33.53 | 27.35 | 27.7 | Positive |
| 5 | No Ct | No Ct | No Ct | No Ct | No Ct | No Ct | No Ct | No Ct | Negative |
| 6 | No Ct | No Ct | No Ct | No Ct | No Ct | No Ct | No Ct | No Ct | Negative |
| 7 | No Ct | No Ct | No Ct | No Ct | No Ct | No Ct | No Ct | No Ct | Negative |
| 8 | No Ct | No Ct | No Ct | No Ct | No Ct | No Ct | No Ct | No Ct | Negative |
| 9 | No Ct | No Ct | No Ct | No Ct | No Ct | No Ct | No Ct | No Ct | Negative |
| 10 | 23.14 | 21.98 | 26.61 | 22.26 | 26.61 | 26.95 | 21.98 | 22.26 | Positive |
| 11 | 28.79 | 27.35 | 33.11 | 27.7 | 33.11 | 33.53 | 27.35 | 27.7 | Positive |
| 12 | No Ct | No Ct | No Ct | No Ct | No Ct | No Ct | No Ct | No Ct | Negative |
| 13 | No Ct | No Ct | No Ct | No Ct | No Ct | No Ct | No Ct | No Ct | Negative |
| 14 | 27.69 | 26.31 | 31.84 | 26.64 | 31.84 | 32.25 | 26.31 | 26.64 | Positive |
| 15 | 25.28 | 23.89 | 32.91 | 20.98 | 22.28 | 23.4 | 24.78 | 22.58 | Positive |
| 16 | No Ct | No Ct | No Ct | No Ct | No Ct | No Ct | No Ct | No Ct | Negative |
| 17 | No Ct | No Ct | No Ct | No Ct | No Ct | No Ct | No Ct | No Ct | Negative |
| 18 | No Ct | No Ct | No Ct | No Ct | No Ct | No Ct | No Ct | No Ct | Negative |
| 19 | 23.98 | 22.78 | 27.58 | 23.07 | 27.58 | 27.93 | 22.78 | 23.07 | Positive |
| 21 | 27.54 | 26.16 | 31.67 | 26.5 | 31.67 | 32.07 | 26.16 | 26.5 | Positive |
| 22 | No Ct | No Ct | No Ct | No Ct | No Ct | No Ct | No Ct | No Ct | Negative |
| 23 | No Ct | No Ct | No Ct | No Ct | No Ct | No Ct | No Ct | No Ct | Negative |
| 24 | 26.218 | 24.33 | 23.3 | 24.8 | 20.62 | 22.05 | 24.33 | 24.8 | Positive |
| 25 | No Ct | No Ct | No Ct | No Ct | No Ct | No Ct | No Ct | No Ct | Negative |
| 26 | 30.5 | No Ct | No Ct | 30.98 | No Ct | No Ct | 32.54 | 29.57 | Negative/ Positive |
| 27 | No Ct | No Ct | No Ct | No Ct | No Ct | No Ct | No Ct | No Ct | Negative |
| 28 | 27.272 | 25.6 | 24.76 | 25.55 | 20.93 | 24.15 | 25.6 | 25.55 | Positive |
| 29 | No Ct | No Ct | No Ct | No Ct | No Ct | No Ct | No Ct | No Ct | Negative |
| 30 | No Ct | No Ct | No Ct | No Ct | No Ct | No Ct | No Ct | No Ct | Negative |
| 31 | 23.12 | 21.96 | 26.59 | 22.24 | 26.59 | 26.93 | 21.96 | 22.24 | Positive |
| 35 | 28.02 | 26.62 | 32.22 | 26.96 | 32.22 | 32.63 | 26.62 | 26.96 | Positive |
| 36 | No Ct | No Ct | No Ct | No Ct | No Ct | No Ct | No Ct | No Ct | Negative |
| 37 | No Ct | No Ct | No Ct | No Ct | No Ct | No Ct | No Ct | No Ct | Negative |
| 38 | No Ct | No Ct | No Ct | No Ct | No Ct | No Ct | No Ct | No Ct | Negative |
| 39 | 27.42 | 26.05 | 31.53 | 26.38 | 31.53 | 31.94 | 26.05 | 26.38 | Positive |
| 63 | 31.82 | 30.23 | 36.59 | 30.61 | 36.59 | 37.06 | 30.23 | 30.61 | Positive |
| 69 | 30.07 | 28.57 | 34.58 | 28.93 | 34.58 | 35.02 | 28.57 | 28.93 | Positive |
| 71 | No Ct | No Ct | No Ct | No Ct | No Ct | No Ct | No Ct | No Ct | Negative |
| 72 | No Ct | No Ct | No Ct | No Ct | No Ct | No Ct | No Ct | No Ct | Negative |
| 73 | No Ct | No Ct | No Ct | No Ct | No Ct | No Ct | No Ct | No Ct | Negative |
| 74 | 26.3 | 24.99 | 30.25 | 25.3 | 30.25 | 30.63 | 24.99 | 25.3 | Positive |
| 77 | 26.15 | 24.84 | 30.07 | 25.16 | 30.07 | 30.46 | 24.84 | 25.16 | Positive |
| 79 | 26.54 | 25.21 | 30.52 | 25.53 | 30.52 | 30.91 | 25.21 | 25.53 | Positive |
| 82 | 28.37 | 26.95 | 32.63 | 27.3 | 32.63 | 33.04 | 26.95 | 27.3 | Positive |
| 84 | 29.58 | 28.1 | 34.02 | 28.46 | 34.02 | 34.45 | 28.1 | 28.46 | Positive |
| 86 | 25.77 | 24.48 | 29.64 | 24.79 | 29.64 | 30.01 | 24.48 | 24.79 | Positive |
| 87 | No Ct | No Ct | No Ct | No Ct | No Ct | No Ct | No Ct | No Ct | Negative |
| 89 | 26.15 | 24.84 | 30.07 | 25.16 | 30.07 | 30.46 | 24.84 | 25.16 | Positive |
| 91 | 27.69 | 30.41 | 33.31 | 30.8 | 31.79 | 35.16 | 30.03 | 30.41 | Positive |
| 92 | No Ct | No Ct | No Ct | No Ct | No Ct | No Ct | No Ct | No Ct | Negative |
| 94 | 27.65 | 26.27 | 31.8 | 26.6 | 31.8 | 32.2 | 26.27 | 26.6 | Positive |
| 95 | No Ct | No Ct | No Ct | No Ct | No Ct | No Ct | No Ct | No Ct | Negative |
| 98 | 30.62 | 29.09 | 35.21 | 29.46 | 35.21 | 35.66 | 29.09 | 29.46 | Positive |
| 99 | 30.51 | 28.98 | 35.09 | 29.35 | 35.09 | 35.53 | 28.98 | 29.35 | Positive |
| 1 | No Ct | No Ct | No Ct | No Ct | No Ct | No Ct | No Ct | No Ct | Negative |
| 2 | No Ct | No Ct | No Ct | No Ct | No Ct | No Ct | No Ct | No Ct | Negative |
| 3 | 23.14 | 25.56 | 33.25 | 25.89 | 32.83 | 36.99 | 25.24 | 25.56 | Positive |
| 8 | 28.79 | 25.3 | 33.31 | 25.62 | 32.89 | 36.26 | 24.98 | 25.3 | Positive |
| 9 | No Ct | No Ct | No Ct | No Ct | No Ct | No Ct | No Ct | No Ct | Negative |
| 10 | No Ct | No Ct | No Ct | No Ct | No Ct | No Ct | No Ct | No Ct | Negative |
