# Supplementary material for: M gene targeted qRT-PCR approach for SARS-CoV-2 virus detection
Source: Sci Rep. 2023 Oct 3;13:16659. doi: 10.1038/s41598-023-43204-9 (PMC10547753; doi:10.1038/s41598-023-43204-9)
Supplement: Supplementary file 2 — Supplementary Table 2. [file 41598_2023_43204_MOESM2_ESM.docx]

**Supplementary File 02**

**Evaluation of different SARS-CoV-2 variant detection using BCSIR-COVID Kit**

| Accession number | Variant | Ct Value |
| --- | --- | --- |
| ON909199 | Delta | 28.02 |
| ON909200 | Delta | 33.62 |
| ON909201 | Delta | 23.14 |
| ON909202 | Delta | 28.79 |
| ON909203 | Delta | 27.69 |
| ON909204 | Delta | 27.54 |
| ON909205 | Delta | 23.12 |
| ON810538 | Omicron | 25.28 |
| ON810539 | Omicron | 26.93 |
| ON810540 | Omicron | 25.21 |
| ON810541 | Omicron | 28.84 |
| ON810542 | Omicron | 27.3 |
| ON810543 | Omicron | 28.37 |
| ON810544 | Omicron | 26.18 |
| ON980705 | Omicron | 30.51 |
| ON980706 | Omicron | 25.77 |
| MZ020420 | Gamma | 28.02 |
| MZ562489 | Beta | 33.62 |
| MZ562480 | Beta | 23.14 |
| MZ562483 | Beta | 28.79 |
| MZ562484 | Beta | 27.69 |
| MZ562485 | Beta | 27.65 |
| MZ562486 | Beta | 30.62 |
| MZ562488 | Beta | 30.51 |
| ON911821 | Alpha | 28.37 |
| ON911822 | Alpha | 29.58 |
| ON911823 | Alpha | 25.77 |
| ON911824 | Alpha | 26.15 |
| ON911825 | Alpha | 26.3 |
| ON911826 | Alpha | 26.15 |
