# Supplementary material for: M gene targeted qRT-PCR approach for SARS-CoV-2 virus detection
Source: Sci Rep. 2023 Oct 3;13:16659. doi: 10.1038/s41598-023-43204-9 (PMC10547753; doi:10.1038/s41598-023-43204-9)
Supplement: Supplementary file 3 — Supplementary Figures. [file 41598_2023_43204_MOESM3_ESM.docx]

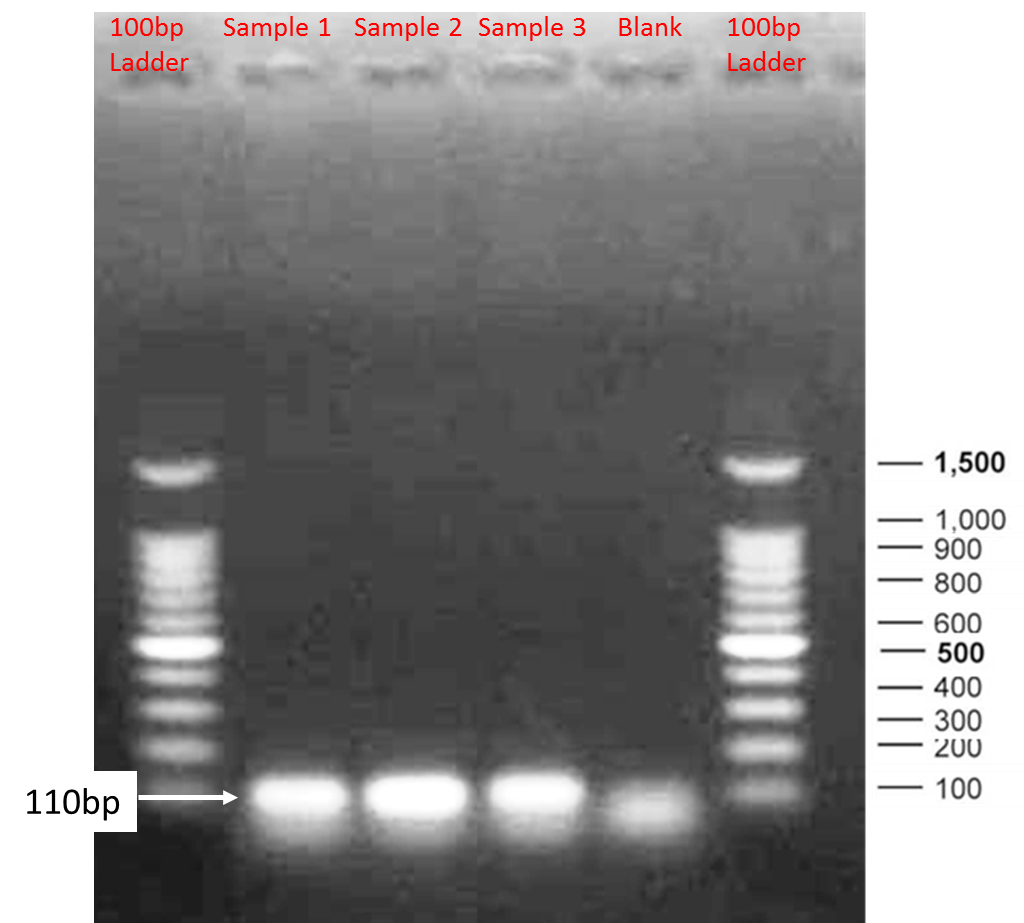


Figure 1: Gel electrophoresis image showing representative 110bp bands for M gene primers. The experiment was done using three randomly picked COVID-19 positive samples and nuclease free water was used as Blank. Tiangen® 100bp DNA Ladder was used as marker.


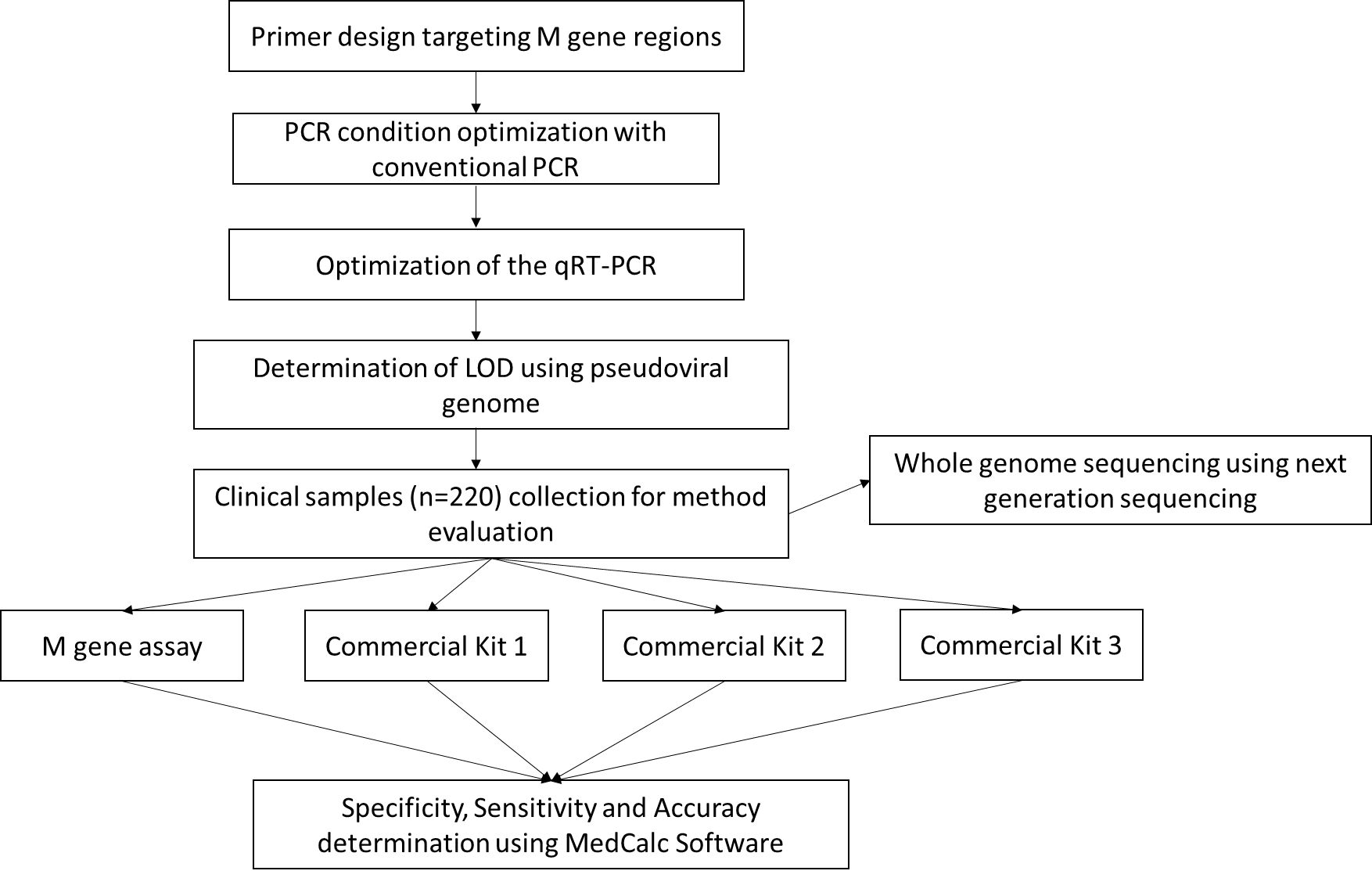


Figure 2: A schematic flow chart showing the overall tests untaken to evaluate the sensitivity and specificity of the M gene assay.
